# Model of zonular forces on the lens capsule during accommodation

**DOI:** 10.1038/s41598-024-56563-8

**Published:** 2024-03-11

**Authors:** Ronald A. Schachar, Ira H. Schachar, Shubham Kumar, Eitan I. Feldman, Barbara K. Pierscionek, Pamela C. Cosman

**Affiliations:** 1 Department of Physics, University of Texas at Arlington, Arlington, TX USA; 2 North Bay Vitreoretinal Consultants, Santa Rosa, CA USA; 3 Department of Electrical and Computer Engineering, University of California San Diego, La Jolla, CA USA; 4 Rice University, Houston, TX USA; 5 Faculty of Health, Medicine and Social Care, Medical Technology Research Centre, Anglia Ruskin University, Chelmsford, UK

**Keywords:** Medical research, Experimental models of disease

## Abstract

How the human eye focuses for near; i.e. accommodates, is still being evaluated after more than 165 years. The mechanism of accommodation is essential for understanding the etiology and potential treatments for myopia, glaucoma and presbyopia. Presbyopia affects 100% of the population in the fifth decade of life. The lens is encased in a semi-elastic capsule with attached ligaments called zonules that mediate ciliary muscle forces to alter lens shape. The zonules are attached at the lens capsule equator. The fundamental issue is whether during accommodation all the zonules relax causing the central and peripheral lens surfaces to steepen, or the equatorial zonules are under increased tension while the anterior and posterior zonules relax causing the lens surface to peripherally flatten and centrally steepen while maintaining lens stability. Here we show with a balloon capsule zonular force model that increased equatorial zonular tension with relaxation of the anterior and posterior zonules replicates the topographical changes observed during in vivo rhesus and human accommodation of the lens capsule without lens stroma. The zonular forces required to simulate lens capsule configuration during in vivo accommodation are inconsistent with the general belief that all the zonules relax during accommodation.

It is well established since 1801 that a change in lens shape is the basis for accommodation^1^. The generally accepted Helmholtz theory predicts that during accommodation the ciliary muscle contracts causing all the zonules to relax and the lens to round-up with an increase in central thickness and central optical power^2^. However, during accommodation, it has been shown that the peripheral anterior lens surface flattens. Tscherning and Fincham observed that reflections from the central anterior lens surface move together while peripheral reflections move apart during accommodation^3,4^. In addition, using Scheimpflug photography, Dubbelman et al. demonstrated during 8 diopters of accommodation the anterior lens surface becomes more curved while “*the peripheral part of the lens becomes flatter*”^5^*.* Consequently, instead of spherical aberration shifting in the positive direction, as would be expected from rounding up of the lens, spherical aberration universally shifts in the negative direction during accommodation^6^. Peripheral lens surface flattening must be the basis for the negative shift in spherical aberration because MRI measurements demonstrated there is no change in peripheral lens refractive index during accommodation (see Fig. 5c of Khan et al.^7^).

According to Helmholtz’s theory, if all zonular tension is decreased, lens central optical power should increase inducing a myopic shift. When the ciliary muscle was disinserted from 23 cynomolgus monkeys causing relaxation of all the zonules, the cynomolgus monkeys became hyperopic, not myopic, with loss of accommodation^8^. If all zonules relaxed during accommodation, gravity should significantly affect amplitude of accommodation since the lens is much denser than the aqueous and vitreous humors^9^. When young students were placed in the supine or prone position, there was no meaningful change in accommodative amplitude^10^. The only difference found was from the head moving approximately 2 mm closer to the target when the student was in the prone position due to indentation of the forehead skin from the head clamp.

Aqueous and vitreous humors have essentially the same densities of 1.005 $\pm$ 0.012 g/cm^3^ and 1.007 $\pm$ 0.010 g/cm^3^, respectively^9^. In view of these equivalent densities, if gravity caused the lens to move, then when prone the anterior chamber should shallow and comparably deepen when supine. Using an optical low-coherence reflectometry biometer (Lenstar LS 900)^11^, anterior chamber depth was measured when unaccommodated subjects were prone and supine^12^. At baseline there was no difference between prone and supine anterior chamber depths. Over 60 min there was no change in supine anterior chamber depth, but prone anterior chamber shallowed a mean of 50 microns. Aqueous fluid can freely move from the posterior to the anterior chamber and the vitreous face is not strong enough to support the lens. Therefore, a difference over time in intraocular pressure or extraocular variables must be the basis for the difference between prone and supine anterior chamber depths and not movement of the lens. Independent of the basis for this difference, in the unaccommodated state, prone anterior chamber shallowed a mean of 50 microns compared to supine. In a separate study, prone anterior chamber depth was compared to upright (sitting) during voluntary and pharmacologically induced maximum accommodation with the same LS 900 in young subjects (mean age = 20.9 years)^13^. Similar to the prior study, when these subjects were prone and unaccommodated, the anterior chamber shallowed 40 microns compared to when they were upright. To control for the anterior chamber shallowing that occurs when the unaccommodated young subject is prone, the difference between accommodative prone minus upright and unaccommodated prone minus upright should be compared to assess the effect of gravity. When this comparison is made, there was zero and a 10-micron difference for voluntary and drug induced maximum accommodation, respectively; i.e. accommodation did not significantly cause more shallowing of the anterior chamber than when the unaccommodated subject moved from upright to prone (Fig. 3c of Lister et al.^13^). Therefore, it is unlikely that gravity affects lens position during accommodation. This implies that during ciliary muscle contraction zonular tension is not reduced, but actually increased resulting in the observed increase in anterior lens capsule stress^14^ and hydrostatic intra-lenticular pressure^15^.

Consistent with Helmholtz’s predictions, multiple ultrasound biomicroscopic (UBM) and magnetic resonant image (MRI) experiments demonstrated that the equatorial lens diameter decreases during accommodation; however, none of these experiments incorporated proper image registration with non-varying references. Image registration is standard practice in medical imaging to minimize the effects of motion artifacts^16–21^. Typical application of image registration involves determining the parameters of a spatial transformation (e.g. rotation, shift, scaling) that enables precise alignment of corresponding reference features (e.g. landmark points) that are common between two images. This precise alignment allows, for example, one image to be subtracted from another or display one as transparent overlay on the other thereby exposing differences between the compared regions of interest.

Image registration has significantly improved measurement accuracy and detection of organ and tissue changes^18–21^. This is especially important for ophthalmic imaging. Normal eye, head and physiologically induced movements make image registration imperative for ophthalmic image comparisons. Image registration has become standard in OCT of the posterior segment of the eye and has led to significant improvements in resolution. Measurements of change in the retinal nerve fiber layer and central retinal thickness have become more accurate^22,23^. Detection of subtle retinal and choroidal pathologies and disease progression, not visible in the past, are now routinely observed^24–28^.

Image registration with high resolution techniques is a basic requirement for evaluating lens changes during accommodation because the eye converges and cyclotorts even with monocular viewing^29–32^. These eye movements are not random which explains the consistent directional changes observed. The importance of image registration is exemplified by studies that did not incorporate image registration and reported mean scleral thickness changes of 390 $\pm$ 330 microns^33^ and even scleral notching during accommodation^34^. With image registration there were no scleral thickness or scleral configurational changes during accommodation^35^. Similarly, with image registration the cornea does not change shape during accommodation^31,36^.

A UBM in vivo study of anesthetized rhesus monkeys during Edinger Westphal electrically stimulated accommodation found the lens equator moved away from the sclera. This study did not incorporate image registration to control for EW stimulated and other possible extraneous eye movements^37^. When image registration techniques were applied to the same UBM video images, the equator actually moved towards the sclera^38^. Similarly, UBM real-time image registration of in vivo human and rhesus monkey pharmacologically controlled accommodation demonstrated that the lens equator moved toward the sclera^39,40^.

Helmholtz’s theory is based on the intuitive concept that when a circumferential equatorial force is applied to an encapsulated elliptical object containing an incompressible material, it will become thinner with both the peripheral and central surfaces flattening. However, when an elliptical object has an aspect ratio $\le$ 0.6, an equatorial circumferential force causes the peripheral surfaces to flatten, and central surfaces to steepen with an increase in central thickness. The phenomenon has been demonstrated in multiple elliptical objects and proven mathematically^41,42^. This counterintuitive topography occurs as a consequence of minimization of curvature and applies to small displacements that are within the range of physiological total ciliary muscle force. This is the foundation of Schachar’s theory^43,44^. Based on this theory, the accommodative amplitudes of vertebrates can be predicted from their lens aspect ratio^45^.

For both the Helmholtz and Schachar theories all the zonules are applying tension when the eye is unaccommodated (Fig. 1a). During accommodation Helmholtz’s theory predicts all zonules relax, equatorial diameter decreases, peripheral and central surfaces steepen, and central optical power and central thickness increase (Fig. 1b). In contrast, the Schachar theory predicts equatorial zonular tension increases, anterior and posterior zonular tension decreases, equatorial diameter increases, peripheral surfaces flatten, central surfaces steepen, and central optical power and central thickness increase (Fig. 1c and d).

**Figure 1:**
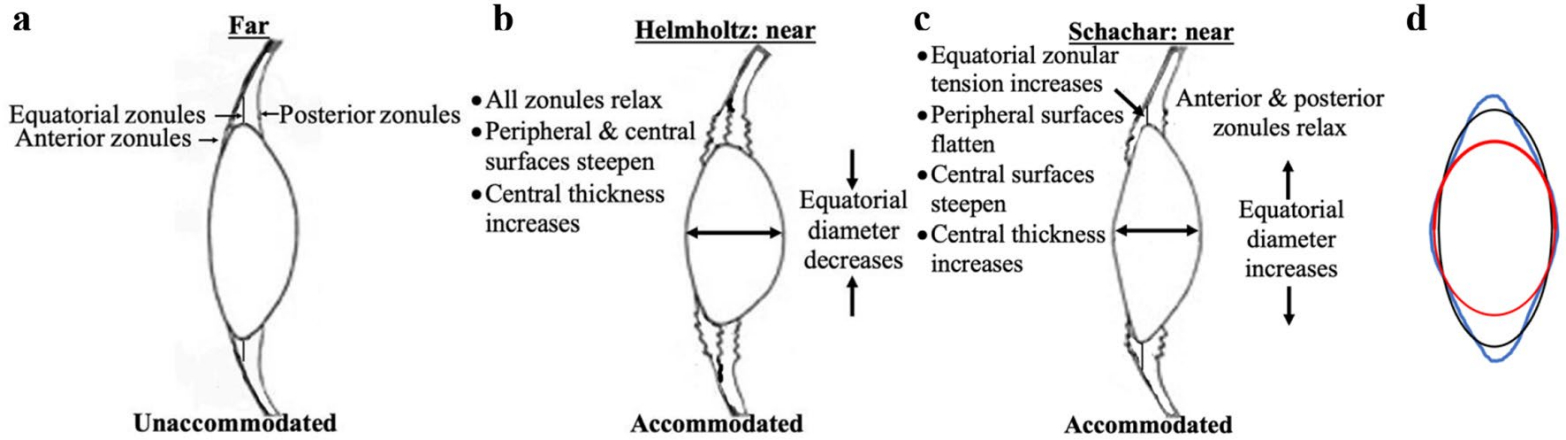
Schematics of the lens. (**a**) At far (unaccommodated), all the zonules are under tension. At near (accommodated) according to (**b**) Helmholtz, all the zonules are relaxed; and therefore, the isolated lens should be maximally accommodated and according to (**c**) Schachar, equatorial zonular tension is increased and the anterior and posterior zonules relax; and therefore, the isolated lens without zonular tension should be unaccommodated. Superimposed representation of lens sagittal profiles when (**d**) unaccommodated (black) and accommodated as predicted by Helmholtz (red) and Schachar (blue).

In agreement with Helmholtz’s theory multiple finite element (FE) analyses have demonstrated lens equatorial diameter decreases during accommodation^46,47^. However, these FE analyses specified the lens nucleus shear modulus is less than that of the lens cortex, which is not appropriate from an understanding of the biochemistry of the lens and in vivo measurements of lens shear modulus. The lens is approximately 40% protein and protein concentration increases from the cortex to the nucleus, which accounts for the lens gradient refractive index^48,49^. Both the higher protein concentration and the presence of more protein disulfide groups indicates that the nucleus must have a shear modulus greater than the lens cortex^50^. This is supported by in vivo Brillouin light scattering that demonstrated the lens nucleus longitudinal bulk modulus, which is directly related to its shear modulus, is markedly greater than the lens cortex at all ages^51,52^. When the lens nucleus shear modulus was specified as the same or greater than the lens cortex, mathematical and FE analyses were consistent with Schachar’s theory while Helmholtz’s theory could only be duplicated by applying forces significantly greater than the maximum force the ciliary muscle can apply^53–55^. For example, to duplicate Helmholtz’s theory when the cortex and nucleus had the same elastic modulus, forces of 0.065 N and 0.12 N^55^, that are greater than the 0.05 N maximum force the ciliary muscle can apply^56^ were required.

One of the major supports for the Helmholtz theory of accommodation is based on topographical lens capsule changes that occurred during accommodation in a 30 y/o patient who had a small hole in the anterior capsule with the rest of the capsule perfectly clear and no vestige of lens matter^57^. Graves noted that when the patient was unaccommodated the anterior and posterior parts of the capsule were fairly smooth as would be expected from both the Helmholtz and Schachar theories with all the zonules applying tension (Fig. 2a). When the patient accommodated, there was increased wrinkling of the central anterior and posterior parts of the capsule (Fig. 2b).

**Figure 2:**
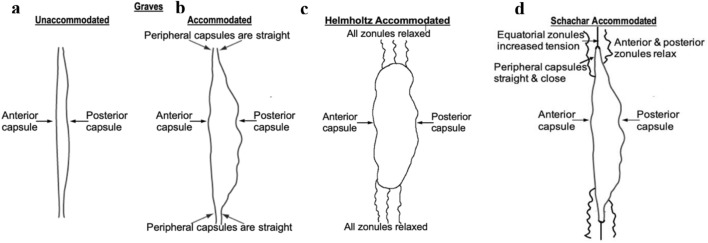
Lens capsule topography. Graves’ drawing of an intact in vivo human lens capsule with no vestige of lens stroma in the (**a**) unaccommodated and (**b**) accommodated states and how they should appear during accommodation according to (**c**) Helmholtz and (**d**) Schachar.

According to Helmholtz’s theory, the capsular wrinkling was due to relaxation of all the zonules and would involve both the central and peripheral anterior and posterior capsular sections (Fig. 2c). However, Graves’ drawing reveals that the peripheral parts of the capsule were straight and close together (Fig. 2b). Similar changes were observed in a rhesus lens capsule during Edington Westphal electrically stimulated accommodation (Fig. 3)^58^. Prior to the EW stimulation the monkey had a lensectomy consisting of removal of the lens stroma by phacoemulsification following an anterior lens capsulotomy.

**Figure 3:**
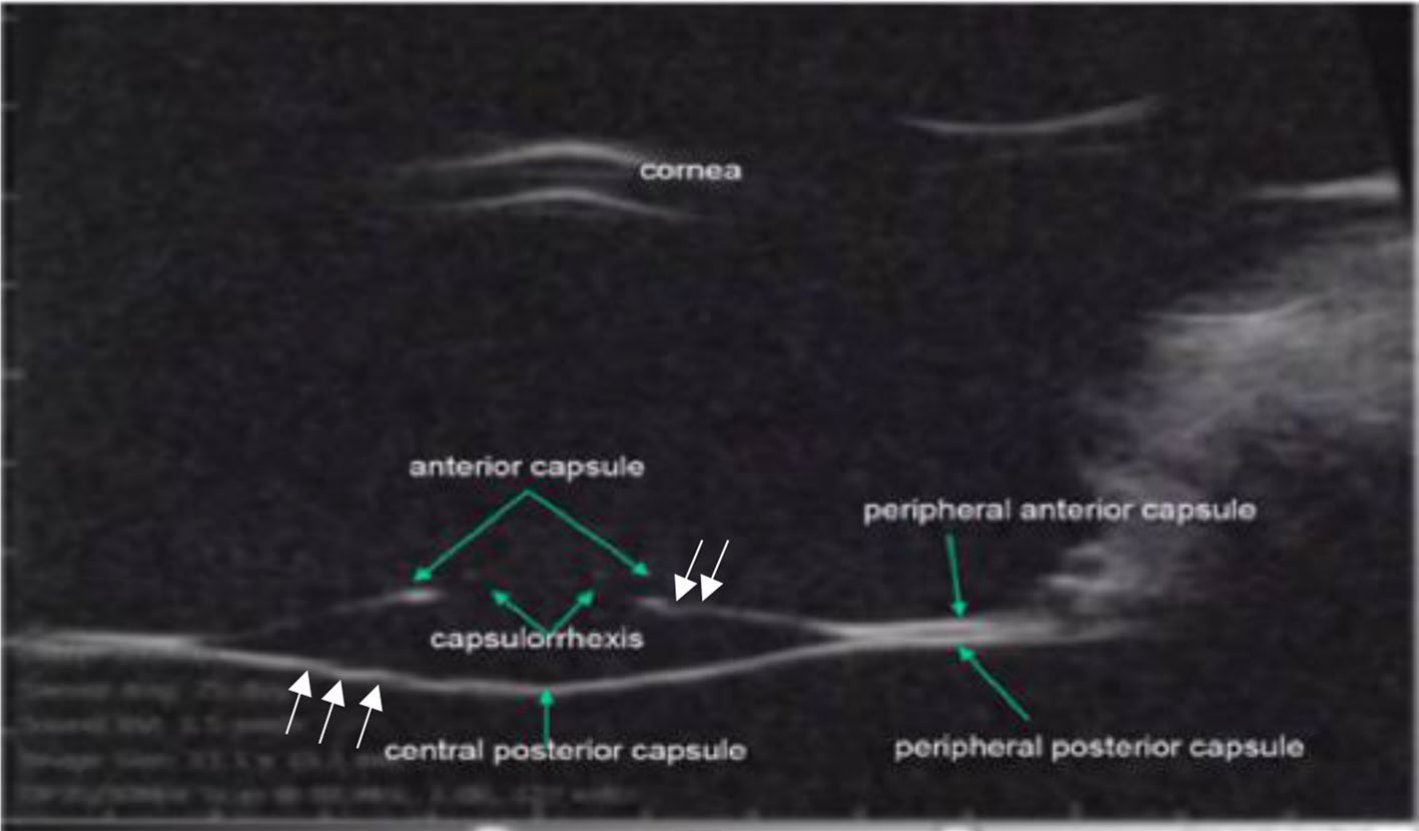
A rhesus monkey lens capsule following extracapsular lens extraction during Edinger Westphal electrically stimulated accommodation (reproduction of from Fig. 6 of Croft et al.^58^). Note the fine capsule wrinkles identified by the added white arrows.

The only way that the peripheral anterior and posterior capsules could straighten and move together while the central anterior and posterior capsular sections were apart and wrinkled was if the equatorial zonules were under tension while the anterior and posterior zonules were relaxed as predicted by Schachar (Fig. 2d).

Ciliary muscle contraction and the qualitative forces transmitted to the lens via the zonules have been shown to be independent of whether the lens stroma is present or not^59^. This was demonstrated in the rhesus monkey following extracapsular lens extraction (ECLE). Movements of the ciliary processes were the same as preoperatively only faster, which is expected because of the reduced mass. Histologically, in all four quadrants of the eyes, the morphology of the ciliary muscle fibers and the ciliary processes were not affected by ECLE. Consequently, it is reasonable to conclude that determining the zonular forces required to replicate the topography of the lens capsule without lens stroma is qualitatively representative of the zonular forces applied during in vivo accommodation. This offers a novel way to analyze zonular forces during accommodation by circumventing the effects of the lens cortical and nuclear shear moduli and lens stroma structure including its contact with the lens capsule. Recent force and FE analyses of the lens capsule without lens stroma found that only when equatorial zonular force was increasing and simultaneously anterior and posterior zonular forces were decreasing can the capsule emulate the in vivo lens capsule topography during accommodation^60^.

To understand how increasing equatorial zonular force with decreasing anterior and posterior zonular force caused the topographical changes, consider a lens capsule with a 1.5 capsulotomy filled with aqueous humor. Equatorial zonular pull on the lens capsule causes the peripheral anterior and posterior capsules to straighten and move together (Fig. 4). The distribution of the equatorial zonular force along the curved lens capsule surfaces generates component anterior and posterior forces directed toward the equatorial capsule axis that move the peripheral lens capsules together (Fig. 4). These component forces directed toward the equatorial axis of the capsule dissipate with distance from the equator of the capsule. Consequently, there is minimal force on the central anterior and posterior capsules directed toward the equatorial axis of the capsule. Since aqueous humor is incompressible, as the anterior and posterior peripheral capsules move together, aqueous humor is forced to move toward the center of the capsule generating outwardly directed forces (Fig. 4). These outwardly directed forces on the central anterior and posterior capsules are significantly greater than the equatorial zonular component forces directed towards the equatorial axis causing the anterior and posterior capsules to move apart and aqueous humor to exit the anterior capsulotomy. Since there is no posterior capsulotomy, the outward force of the aqueous humor causes the posterior capsule to bow posteriorly as observed in vivo (Fig. 4)^61^.

**Figure 4:**
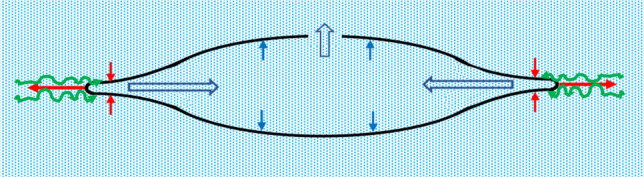
The change in lens capsule shape from a baseline capsule containing aqueous humor when equatorial zonular tension is increased (horizontal red arrows) and anterior and posterior zonular is tension relaxed (green arrows). The equatorial zonular forces are distributed along the curved lens capsule surface inducing component anterior and posterior forces towards the equatorial lens axis (vertical red arrows). Since aqueous humor is incompressible, aqueous humor is forced to move towards the center (large double blue arrows) inducing outward forces causing the central anterior and posterior capsules to move apart (single blue arrows) as aqueous humor exits the anterior capsulotomy and the posterior capsule bows posteriorly.

To validate the force and prior FEM analyses, the present study evaluated zonular forces on a latex balloon model of the lens capsule without lens stroma.

## Methods

To model the unaccommodated and accommodated effects of the zonular forces following an extracapsular lens extraction, a central 5.00 mm trephined hole was cut in the anterior surface of a latex balloon molded from an enlarged wax model of the rhesus monkey lens. The balloon had a central thickness, equatorial diameter and membrane thickness of 12.5 mm, 24 mm, and 0.23 mm, respectively. The aspect ratio (minor axis/major axis) of 0.52 was similar to a 7 y/o rhesus monkey lens^62^.

To represent the anterior, equatorial and posterior zonules, three 6–0 silk sutures were attached (Loctite AA3035, Henkel Corporation, Irvine, CA, USA) symmetrically to the latex balloon equator at 8 equally spaced meridians. To proportionally account for the difference in size, the sutures representing the anterior and posterior zonules were attached 4.5 mm anterior and 3.0 mm posterior to the equatorial suture, respectively^63,64^. A centrally located 0.7 mm hole was drilled into the tabs of stainless-steel thumb screws. The thumb screws were mounted symmetrically on the top edge of a stainless-steel ring that had an outer diameter, inner diameter and height of 139.7 mm, 114.3 mm and 25.4 mm, respectively. Each suture was passed through the central hole in the thumb screw tabs and the device was submerged below the water level of a clear glass container measuring 16 × 16 × 16 cm. The sutures were passed over the sides of the glass container.

Photographs of the latex balloon profile were taken under the following scenarios. First, to simulate baseline unaccommodated rhesus ciliary muscle force^56^ and considering the difference in cross-sectional area and elastic modulus (1.13 MPa)^65^ of the latex balloon compared to the lens capsule (0.034 MPa)^66,67^, 7.0 g lead weights were attached to all the sutures for a total force of 168.0 g. Second, the mechanism of accommodation was emulated by increasing the weights on the equatorial zonules to 22 g (total force = 176 g) and removing the weights from the anterior and posterior zonules to emulate the maximum force the ciliary muscle can apply^56^. Finally, accommodation was simulated as per Helmholtz by removing all zonular weights permitting them to relax. Photographic profiles of the balloon model were compared to the configurations of the unaccommodated and accommodated rhesus and human lens capsule with no lens stroma**.**

## Results

**Figure 5:**
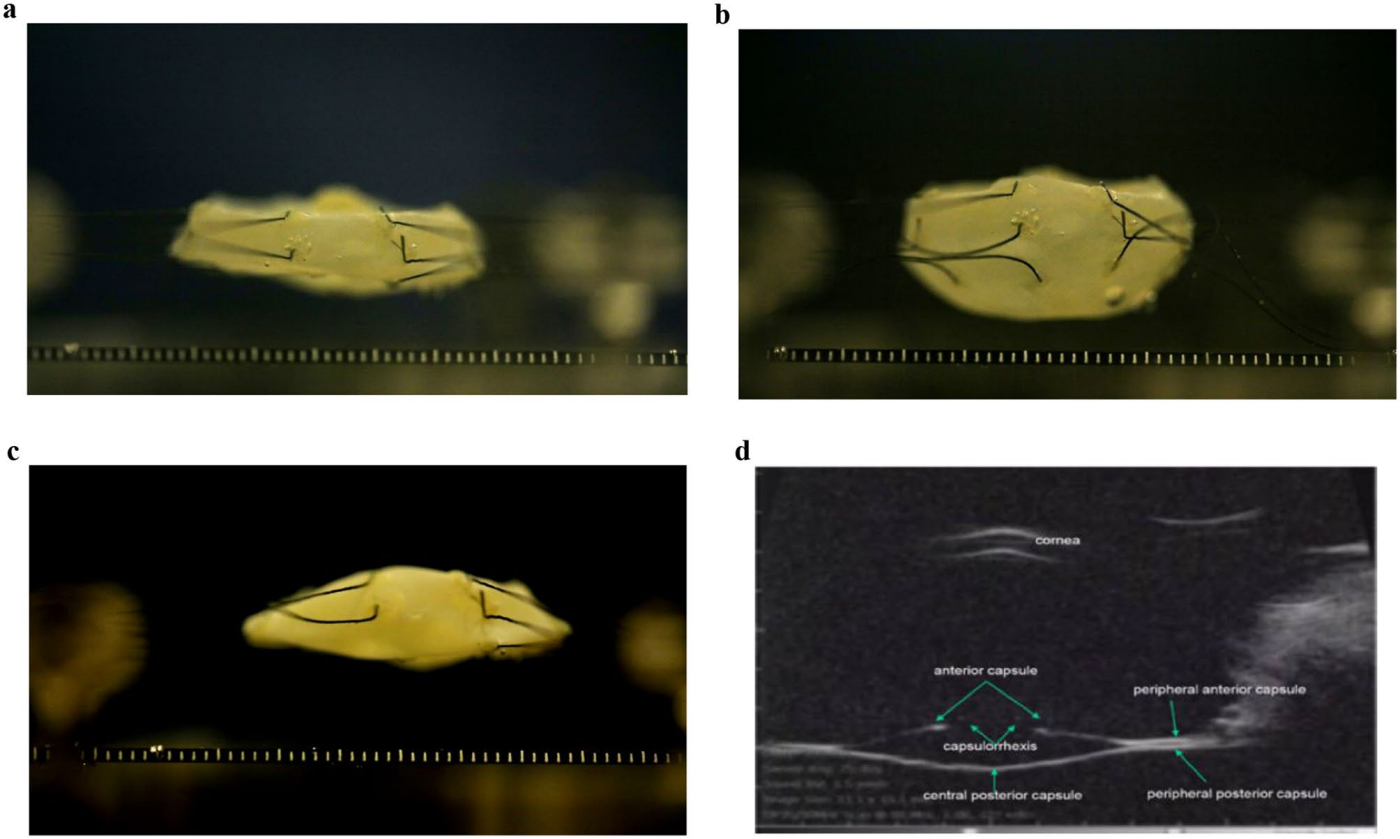
Latex balloon zonular force lens capsule model following an extracapsular lens extraction (anterior capsule up). (**a**) Unaccommodated lens capsule with equal force of 7 g on each of the black 6–0 silk sutures representing the anterior, equatorial and posterior zonules. The accommodated lens capsule according to (**b**) Helmholtz with all the zonules relaxed and according to (**c**) Schachar with 22 g force on each of the equatorial zonules with the anterior and posterior zonules relaxed and (**d**) A rhesus monkey lens capsule following extracapsular lens extraction during Edinger Westphal electrically stimulated accommodation (reproduction from Croft, et al.^58^). Note the similarity in topography of (**c**) and (**d**) with the anterior and posterior peripheral lens capsules close together while the anterior and posterior central capsules are apart.

For the unaccommodated state 7 g weights were applied to each of the black 6–0 silk sutures representing the anterior, equatorial, and posterior zonules for a total equally distributed force of 168 g (Fig. 5a). To emulate the Helmholtz theory all the sutures were relaxed (Fig. 5b). To simulate the Schachar mechanism, a total equatorial suture force of 176.0 g (22 g/equatorial suture) was applied while the anterior and posterior sutures were totally relaxed (Fig. 5c). The topography of these simulations were compared to the rhesus monkey lens capsule following ECLE during Edinger Westphal electrically stimulated accommodation (Fig. 5d).

**Figure 6:**
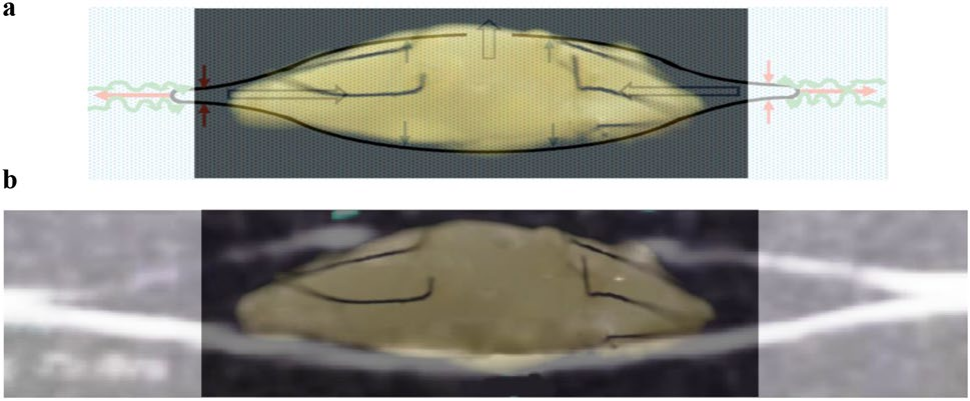
Photograph of the balloon lens capsule model with the sutures representing the equatorial zonules under tension and the sutures representing the anterior and posterior zonules totally relaxed superimposed (**a**) on the schematic of the force analysis when equatorial zonular tension is increased and the anterior and posterior zonules are relaxed (Fig. 4) and (**b**) on the B-scan image of the rhesus lens capsule without lens stroma during accommodation (Fig. 3).

When equatorial zonular force was applied with the anterior and posterior zonules relaxed, the balloon lens capsule model qualitatively replicated the accommodated appearance predicted by the force analysis (Fig. 6a) and the observed topography of the accommodated rhesus lens capsule (Fig. 6b). The peripheral anterior and posterior capsules moved together while the central anterior and posterior capsules moved apart. While there is a remaining discrepancy between the shape of the balloon model and that of the rhesus lens capsule, more force could not be applied to the sutures representing the equatorial zonules to make the balloon model more closely emulate the rhesus lens capsule, because such force caused the sutures to detach from the balloon due to the high elastic modulus of the latex and insufficient strength of the adhesive.

When all the zonules were relaxed, the balloon model appeared relatively elliptical and did not resemble the accommodated rhesus lens capsule (Fig. 5b).

## Discussion

Only when equatorial zonular tension was increased and the anterior and posterior zonules were relaxed did the balloon model emulate the accommodated topography of the in vivo rhesus and human lens capsules. These findings validate the force and FE analyses^60^.

Although the balloon model was an enlargement of the rhesus monkey, rhesus monkeys have served as a reliable model for human eye physiology even though the rhesus monkey ciliary muscle contraction can generate greater central optical power changes than the human^68^. The anatomy, ciliary muscle contraction and zonular forces in the rhesus monkey and human are comparable and both can be interchangeably used to understand the basis for how zonular forces change lens shape^69^.

During accommodation equatorial zonular tension is increased. Equatorial zonules are present throughout life and have a diameter of ~ 10 μm making them clinically not visible^70,71^ In contrast, the anterior and posterior zonules have a diameter of ~ 150 $\mu m$, making them readily visible^63,64,71^. In vivo videography demonstrated that the anterior and posterior zonules relax and actually fold during maximum accommodation during Edinger-Westphal electrically stimulated accommodation in the rhesus monkey^72^. This observation incorrectly implied that all zonules relax during maximum accommodation. As discussed previously, this implication is contradicted by the observed hyperopic refractive shift following total zonular relaxation from ciliary muscle disinsertion^8^, and the increased anterior lens capsule stress^14^ and hydrostatic-lenticular pressure during ciliary muscle contraction^15^. The findings of the present study along with data from image registration studies necessitates re-evaluation of the etiology and potential treatments for myopia, presbyopia, glaucoma and cortical cataracts. For example, for the development of myopia, one hypothesis considers that during accommodation ciliary muscle force is directed inward instead of outward^73^.

Increased equatorial zonular tension with simultaneous anterior and posterior zonular tension reduction occurs from the directional forces generated by contraction of the five functional parts of the ciliary muscle^43,44^. As observed by OCT, the posterior longitudinal and posterior radial ciliary muscle fibers pull the pars plana anteriorly causing the origin of the anterior and posterior zonules to move anteriorly and relax^74^. Simultaneously, contraction of the anterior longitudinal, anterior radial and isometric contraction of the circular muscle fibers causes a notch in the anterior radial muscle fibers moving the origin of the equatorial zonules toward the sclera increasing tension on the equatorial zonules^43,44^. The anterior radial ciliary muscle notch has been predicted by deformation analysis and observed during in vivo accommodation with optical coherence tomography (OCT)^75–77^.

## Conclusions

In the unaccommodated state all the zonules are applying tension. During accommodation the anterior and posterior zonules relax and can even fold during maximum accommodation while equatorial zonular tension increases to induce accommodation and maintain lens stability. These analyses demonstrate that relaxation of all the zonules does not occur during accommodation.

## Data Availability

All data generated or analyzed during this study are included in this published article.
